# Exploring the interplay between running exercises, microbial diversity, and tryptophan metabolism along the microbiota-gut-brain axis

**DOI:** 10.3389/fmicb.2024.1326584

**Published:** 2024-01-22

**Authors:** Alejandra Vazquez-Medina, Nicole Rodriguez-Trujillo, Kiara Ayuso-Rodriguez, Fabiana Marini-Martinez, Roberto Angeli-Morales, Gerardo Caussade-Silvestrini, Filipa Godoy-Vitorino, Nataliya Chorna

**Affiliations:** ^1^Department of Biochemistry, University of Puerto Rico, Medical Sciences Campus, San Juan, Puerto Rico; ^2^Nutrition and Dietetics Program, University of Puerto Rico, Rio Piedras Campus, San Juan, Puerto Rico; ^3^Department of Biology, University of Puerto Rico, Rio Piedras Campus, San Juan, Puerto Rico; ^4^Department of Microbiology and Medical Zoology, University of Puerto Rico, Medical Sciences Campus, San Juan, Puerto Rico

**Keywords:** tryptophan, exercise, microbiota, gut, brain, taxon-function

## Abstract

The emergent recognition of the gut-brain axis connection has shed light on the role of the microbiota in modulating the gut-brain axis’s functions. Several microbial metabolites, such as serotonin, kynurenine, tryptamine, indole, and their derivatives originating from tryptophan metabolism have been implicated in influencing this axis. In our study, we aimed to investigate the impact of running exercises on microbial tryptophan metabolism using a mouse model. We conducted a multi-omics analysis to obtain a comprehensive insight into the changes in tryptophan metabolism along the microbiota-gut-brain axis induced by running exercises. The analyses integrated multiple components, such as tryptophan changes and metabolite levels in the gut, blood, hippocampus, and brainstem. Fecal microbiota analysis aimed to examine the composition and diversity of the gut microbiota, and taxon-function analysis explored the associations between specific microbial taxa and functional activities in tryptophan metabolism. Our findings revealed significant alterations in tryptophan metabolism across multiple sites, including the gut, blood, hippocampus, and brainstem. The outcomes indicate a shift in microbiota diversity and tryptophan metabolizing capabilities within the running group, linked to increased tryptophan transportation to the hippocampus and brainstem through circulation. Moreover, the symbiotic association between *Romboutsia* and *A. muciniphila* indicated their potential contribution to modifying the gut microenvironment and influencing tryptophan transport to the hippocampus and brainstem. These findings have potential applications for developing microbiota-based approaches in the context of exercise for neurological diseases, especially on mental health and overall well-being.

## 1 Introduction

Evidence suggests that microbiota-mediated brain plasticity is linked to metabolites produced by the gastrointestinal tract (gut) microbiota, which respond to variations in the extrinsic environment. Factors such as intermittent bioenergetic challenges like exercise, diet, and intellectual stimulation-rich lifestyles have promoted healthy brain functioning (Finlay et al., 2019; Fang et al., 2022). Conversely, a sedentary and overindulgent lifestyle can lead to metabolic dysregulation, memory loss, and neurodegenerative diseases (Catena-Dell’osso et al., 2013). The gut microbiota is sensitive to variations in the extrinsic environment and can alter its structure and metabolic activity, impacting bidirectional communication along the gut-brain axis.

Recent studies in basic and clinical research have highlighted the link between gut microbiota and tryptophan (TRP) metabolism disturbances. Our research investigates the increasing effects of voluntary running exercise on alterations in TRP metabolism along the gut-brain axis and the involvement of the gut microbiota in this process. TRP, an essential amino acid obtained from the diet, is a precursor for serotonin, kynurenine, indole, and their derivatives, which could play crucial roles in regulating brain functions (Liaqat et al., 2022). Approximately 90% of the digested TRP is metabolized along the kynurenine axis (Badawy, 2017), while only 3% is metabolized to serotonin throughout the body. About 7% of TRP is degraded via the indole pathway by Firmicutes, Bacteroidetes, and Proteobacteria (Richard et al., 2009; Badawy, 2017). In contrast to TRP and kynurenine, serotonin does not cross the blood–brain barrier (BBB). Studies suggest that indole can also cross the BBB and exhibit pro-inflammatory activity by activating the aryl hydrocarbon receptor pathway in the astrocytes (Ojo and Tischkau, 2021; Zhou et al., 2021; Huang et al., 2023). Two major pools of serotonin are recognized: 95% is the gut serotonin required for the regulation of intestinal homeostasis and predominantly produced by *Clostridial* species (Yano et al., 2015; Fung et al., 2019), and nearly 5% corresponds to the serotonin produced by the host brain from TRP and serotonin (Gostner et al., 2020).

Intense competition exists between serotonin, indole, and kynurenine for the available TRP. Under certain pathological conditions or unfavorable changes in the lifestyle, more indole and kynurenine, including their metabolites, are produced at the expense of serotonin. Under certain pathological conditions or unfavorable lifestyle changes, an increased production of indole and kynurenine, along with their metabolites, occurs at the expense of serotonin. This phenomenon has been observed in humans (Keszthelyi et al., 2012), rats (Gál and Sherman, 1980), and simulated conditions *in silico* (Kaur et al., 2019). Moreover, this metabolic shift takes place in the brain, leading to behavioral changes, including persistent sadness and loss of interest, frequently noted in patients diagnosed with psychological and neurodegenerative disorders, as reported by *in silico* approach (Catena-Dell’osso et al., 2013). As indicated by findings from human studies, elevated levels of kynurenine in the brain have been proposed to play a role in the pathophysiology of suicidal behavior (Sublette et al., 2011; Wu et al., 2018), as well as in elderly dementia patients (Van Der Velpen et al., 2019; González-Sánchez et al., 2020).

Running exercises have been recognized as vital for human health and have demonstrated cognitive benefits. As the global population ages and cognitive dysfunction becomes more prevalent, non-pharmacological interventions like running exercises have proven effective in enhancing cognitive function (Chen et al., 2022). The regulation of serotonin and other mechanisms are believed to contribute to these mental effects. However, the complex molecular and metabolic mechanisms underlying the cognitive benefits of exercise, particularly TRP metabolism and the gut-brain axis, still need to be completed. Additionally, studies have found that the gut microbiota is associated with greater feelings of happiness and hopefulness, including lower levels of Firmicutes *bacterium CAG 94* or *Ruminococcaceae bacterium D16* (Ke et al., 2023). Therefore, the objective of our study was to investigate the effects of voluntary running exercise on alterations in TRP metabolism along the microbiota-gut-brain axis, with a specific focus on the association of the gut microbiota with TRP metabolism. Our aims included identifying the impact of running exercises on overall gut microbial communities (assessing both alpha and beta diversity estimates) and its endogenous capacity to modulate TRP metabolism in the host.

Additionally, we aimed to decipher the influence of running activity on microbiota/host metabolome interactions that regulate peripheral and neuronal TRP metabolism. Through these investigations, we predicted a systemic network connecting the brain and gut. This preliminary may contribute to developing novel therapeutic approaches for psychiatric disorders and neurodegenerative diseases, ultimately improving the overall well-being of individuals.

## 2 Materials and methods

### 2.1 Mice model of voluntary running exercise

A voluntary running exercise model involving mice to investigate changes in TRP was assessed and approved by the University of Puerto Rico Institutional Animal Care and Use Committee (IACUC) under protocol number A660121. We specifically opted for voluntary wheel running exercise instead of forced treadmill running, as it closely mimics the natural running behavior of mice without inducing stress or interference from the research, as previously reported by Manzanares et al. (2018). To evaluate our hypothesis that running exercise reshapes gut microbiota diversity that balances TRP metabolism in the gut, we used an established model of voluntary running exercise in 20 weeks-old male mice (C57BL/6 J, Jackson Lab) housed individually in a standard cage in temperature-controlled (21°C) quarters with a 12 h light/12 h dark cycle. Animals were given water and food (Purina Chow) *ad libitum* as previously described (Chorna et al., 2013). Briefly, mice were randomized into two groups, sedentary control (SED, *n* = 12) and running experimental (RUN, *n* = 12), housed with free access to a wireless running wheel (Med Associates) for 42 days. For the SED group, the wheels were locked in the experiment. Therefore, this group of mice could not perform running exercises. Running activities of the RUN group were recorded for each animal for the investigation to ensure that each mouse in the RUN group was physically active. The recording was conducted using an automatic counter and Med Associates software. The mice engaged in running sessions daily from 6:00 pm to 5:00 am, maintaining an average running speed of 4 km/h.

### 2.2 Extraction of fecal metabolites

50 mg of fresh feces were collected aseptically from each mouse and frozen in liquid nitrogen. For Gas Chromatography/Mass Spectrometry (GC/MS) analysis, samples were homogenized in 800 μL of the chloroform methanol-water (v/v 2:5:2) solution, vortexed for 10 min at 4°C, and centrifuged at 14 K rpm × 10 min at 4°C. The supernatant was collected and evaporated to dryness under a nitrogen gas stream at 50°C (RapidVap, Labconco) and stored at −80°C. For Liquid Chromatography/Mass Spectrometry (LC/MS) analysis, 50 mg of fresh feces samples were spiked with 0.5 mL water, homogenized for 5 min followed by 10 s of sonication, and centrifuged at 14 K rpm × 10 min at 4°C (Shan et al., 2018). Collected supernatants were evaporated to dryness under a nitrogen gas stream and stored at −80°C.

### 2.3 Extraction of the whole blood metabolites

Blood samples were collected postmortem from the trunk after decapitation, sonicated by three strokes on ice using the Sonic Dismembrator (Fisher Scientific). From each sample, 130 μL of blood were placed in the new tube with 100 μL of Mammalian Protein Extraction Reagent (MPER, Thermo Fisher Sci), followed by 20 s vortexing and fast spin on table centrifuge at RT. Supernatants were collected and deproteinized by centrifugation at 4,6 K rpm × 30 min using Vivaspin^®^ 500 (Sigma-Aldrich). For GC/MS analysis: 800 μL of the chloroform methanol-water (v/v 2:5:2) was added to each eluate, centrifuged at 14 K rpm × 10 min at 4°C, evaporated to dryness under a nitrogen gas stream and stored at −80°C.

### 2.4 Extraction of brain metabolites

Mouse brains were dissected and washed with ice-cold saline for 30 s to remove excess blood. Hippocampus and brainstem samples were rapidly isolated, quenched in liquid nitrogen and weighed (Chorna and Chornyy, 2019). The samples were subsequently stored at −80°C. The processing of frozen brain regions followed the procedures outlined in a prior publication (Miller et al., 2006). Briefly, approximately 20–40 mg of frozen tissues were resuspended in 4 volumes of MPER and homogenized on ice. 130 μL of tissue homogenates were spin filtered, as above, and the flow through was diluted 1:1 with deionized water, followed by sonication for 10 s using a Sonic Dismembrator on ice, vortexed for 10 s, and centrifuged at 14 K rpm × 15 min at 4°C. For GC/MS analysis: 800 μL of the chloroform methanol-water (v/v 2:5:2) was added to 100 μL of eluate, centrifuged at 14 K rpm × 10 min at 4°C, evaporated to dryness under a nitrogen gas stream and stored at −80°C. For LC/MS analysis: 100 μL of eluate was evaporated to dryness under a nitrogen gas stream and stored at −80°C.

### 2.5 Derivatization of samples for GC/MS analysis

Fecal, blood, hippocampus, and brainstem samples from the SED and RUN groups underwent two steps of derivatizations. Briefly, dried samples were first derivatized by methoxyamination by adding 30 μL of 20 mg/mL methoxyamine hydrochloride solution in pyridine (Sigma-Aldrich) and incubated at 37°C for 2 h. Trimethylsilylation was subsequently performed by adding 30 μL of N-methyl-N-trimethylsilyl-trifluoroacetamide (MSTFA + 1% TMCS, Sigma-Aldrich) and incubated for 1 h at 65°C. Samples were centrifuged at 14 K rpm for 10 min at RT. Supernatants were transferred to glass vials. 20 μL of each sample was added to analytical glass vials with inserts and processed by GCMS/MS-TQ8050 (Shimadzu Inc.) using analytical conditions as previously described (Chornyy et al., 2017; Chorna and Chornyy, 2019). Briefly, the chromatography conditions were as follows: RXI-5MS (0.25 mm inner diameter, 0.25 μm D.F., 30 m) (Restek), split injection (ratio = 15), injection volume of 1 μL. Inlet temperature was 280°C; ion source temperature was 200°C; interface temperature was 150°C. The oven temperature was set at 100°C for 1 min, and then programmed from 100°C to 290°C at 8°C/min, held at 290°C for 16 min. Helium was the carrier gas at a constant linear velocity of 39 cm/s. MS conditions: electrospray ionization (ESI) source, full scan mode, electron energy of 70 eV, quadrupole scan range of m/z 35–700. Quality assessment and quality controls were accomplished by employing several blank samples, including system suitability blanks and derivatization processing blanks. To mitigate systematic bias, the order of sample analysis was randomized, and blanks and quality control samples were evenly distributed among the injections to monitor instrument stability.

### 2.6 GC/MS raw data analysis

The GC/MS raw data obtained from fecal, blood, hippocampus, and brainstem samples were processed using the Labsolution Postrun analysis software (Shimadzu Inc.) equipped with NIST14/2014/EPA/NIH database to identify the metabolites. Following peak integration through the Labsolution Postrun analysis software and multiple searches in the mass spectral library database.

### 2.7 LC/MS raw data analysis

The LC/MS experiments were outsourced and conducted at the Proteomics, Metabolomics, and Mass Spectrometry Facility at the Montana State University (MSU), MT, using an i-Class LC system coupled to a Synapt-XS mass spectrometer (Waters). Liquid chromatography was performed on fecal, hippocampus, and brainstem samples collected from the SED and RUN groups. The chromatographic separation was conducted using a BEH HILIC column with 1.7 μm particle size dimensions and 2.1 × 100 mm (Waters). The column temperature was maintained at 50°C, and the flow rate was set at 0.4 mL/min. The mobile phase consisted of 0.1% formic acid in water as solvent A and 0.1% formic acid in acetonitrile as solvent B. The initial mobile phase composition was 5% A and 95% B, held constant for 2 min. Subsequently, a linear gradient from 5 to 50% A was applied over 8 min, followed by a 2 min hold at 50% B before returning to the initial condition for a 5 min re-equilibration. The total run time for each analysis was 19 min. The LC eluent was connected to an electrospray ionization source operating in the positive-ion mode. The mass spectrometer operated at a frequency of 5 Hz, covering a mass range of 50–1,200 m/z. The LC-MS raw data files (.raw) were converted to the mzML format using MSConvert (Chambers et al., 2012), and XCMS (Tautenhahn et al., 2012) was used for feature alignment and extraction. MS^E was performed on a pooled sample, and identifications were made using Progenesis. A total of 1,554 spectral features were detected in fecal, hippocampus, and brainstem samples collected from both the SED and RUN groups. These features were identified based on their mass-to-charge ratio (m/z) and retention time in chromatography. Before identification, the data underwent quality control filtering and were normalized to weight using MetaboAnalyst 5.0. We next performed a functional enrichment analysis using the MS peaks tool of MetaboAnalyst 5.0, which incorporates the mummichog algorithm (Li et al., 2013; Pang et al., 2022). This algorithm utilizes a KEGG database of identified and quantified empirical compounds to infer metabolic pathway activity from LC/MS metabolomics data. The Mummichog algorithm has been shown to perform superior to other pathway enrichment methods for functional interpretation of LC/MS data (Lu et al., 2023a). The functional enrichment analysis was conducted with a mass tolerance of 10 ppm. Primary ions were considered valid to reduce the number of significantly altered empirical compounds. The selected library for analysis was the KEGG database of the *Mus musculus* species. For the Mummichog algorithm, we set a value of *p* cut-off of 0.15 (default top: 10% peaks). A list of probable TRP metabolite identifications related to kynurenine, indole, and serotonin pathways was generated from the pathway enrichment analysis.

### 2.8 Differential analysis of identified metabolites

To evaluate significant differences in TRP levels detected by GC/MS and TRP derivatives detected by LC/MS within both the SED and RUN groups, the corresponding peak intensities for each sample were acquired and processed using MetaboAnalyst 5.0 (Pang et al., 2021, 2022). Briefly, data collected from fecal and brain samples were subsequently normalized by weight. Data from blood samples were normalized to the volume used for analysis, and all data were subjected to log transformation and range scaling. We next conducted a statistical analysis using the Mann–Whitney U test, and the resulting *p*-values were adjusted for multiple comparisons using the false discovery rate (FDR) method. A significance level (*α*) of 0.05 was applied to determine statistical significance. The “*p*_adj” (adjusted *p* value) abbreviation was used to distinguish from the “*p*” unadjusted *p* value across the manuscript and figures. Bar plots were generated using GraphPad Prism version 10.0.3 (GraphPad Software).

### 2.9 Processing of 16S rRNA reads and quality control

Fecal samples collected from two groups, SED (*n* = 12) and RUN (*n* = 12), underwent genomic DNA extractions using the Qiagen Power Soil Kit (QIAGEN LLC, United States), following a previously optimized protocol outlined in our study (García-Amado et al., 2023), which consisted of the original manufacturer’s protocol with the binding step, only 400 μL of the lysate (lysate + 100%ethanol + C4) were added to the spin filter column at a time and bind in consecutive steps through a vacuum manifold until all the lysate was bound to the membrane. Elution was done with 100 μL sterile PCR water. The extracted DNA was quantified using the Qubit^®^ dsDNA HS (High Sensitivity) Assay (Waltham, United States), with 5–100 ng/μL concentrations. An average of 10–30 ng/μL of genomic DNA was sent to an external sequencing facility. The V4 hypervariable region of the 16S ribosomal RNA gene (~291 bp) was amplified using universal bacterial primers (515F, 5′GTGCCAGCMGCCGCGGTAA3′ and 806R, 5′GGACTACHVGGGTWTCTAAT3′), following previously reported conditions from our group’s projects (Rodríguez-Barreras et al., 2021; Ruiz Barrionuevo et al., 2022) within the Earth Microbiome Project (Caporaso et al., 2012). Sequencing of the 16S amplicons was conducted using the Illumina MiSeq Reagent kit 2 × 250 bp (outsourced). The 16S-rRNA forward and reverse reads, along with the corresponding metadata file, were deposited in the QIITA Bio project ID 15005 (Gonzalez et al., 2018). In QIITA, forward reads were pre-processed by demultiplexing using split libraries FASTQ in QIITA with default parameters and a Phred offset 33. Trimming was performed to 250 bp. OTU picking used a closed reference approach with the SILVA reference database for taxonomy assignment, setting a minimum similarity threshold of 97% (Pruesse et al., 2007).

### 2.10 Statistical analyses of sequence data and visualizations

From QIITA, the resulting species table file in biom format was downloaded for downstream analyses using a locally run version of QIIME2 (Bolyen et al., 2019). A total of 896 features remained after excluding sequences matching chloroplasts, mitochondria, and taxonomically unassigned sequences, as well as singleton amplicons. As a result, the dataset comprises 510 features, each having a minimum of 12,826 reads, with a rarefaction level set at 20,000. Further data analyses were performed using MicrobiomeAnalyst 2.0 (Lu et al., 2023b). Data was scaled using the total sum scaling (TSS) method. Community profiling was performed by measuring alpha diversity, including richness (Chao 1 and Observed), ACE (Abundance-based Coverage Estimator), and Fisher diversity using the Mann–Whitney U test. In addition, the figures were generated using GraphPad Prism version 10.0.3 (GraphPad Software) based on the data obtained from MicrobiomeAnalyst for each analysis. Nonparametric statistical *t*-tests with Monte Carlo permutations were performed to assess the significance of differences between groups. For community-level analyses (beta diversity), pairwise Bray–Curtis distances were calculated between samples. The statistical significance between sample groups (beta diversity) was evaluated using the PERMANOVA (Permutational Multivariate Analysis of Variance) test (Anderson, 2001). To discriminate between the SED and RUN groups and identify significant features, Linear Discriminant Analysis Effect Size (LEfSe) was employed with a value of *p* cut-off of 0.05, FDR-adjusted value of *p* cut-off 0.1, and a Log LDA Score threshold of 5.0 (Segata et al., 2011). Sparse Correlations for Compositional data (SparCC) (Friedman and Alm, 2012) were used to calculate correlation coefficients of relative abundance and create a network of co-abundant species at the genus level (Permutation = 100, value of *p* threshold = 0.05, correlation threshold = 0.4). Changes in the bacterial interaction network were illustrated by variations in positive and negative correlations observed among the taxa. In our study, we interpreted positive correlations (co-abundance) as indicative of potential symbiosis, while negative correlations (co-exclusion) were considered potentially antagonistic. This interpretation establishes a meaningful framework grounded in the ecological context of the microbial communities under investigation. A recent study by Metcalfe et al. (2021) confirms that SparCC correlation analysis can be a valuable tool for identifying microbial interactions and likely symbioses in various environments. During our investigation, we considered the proximity and coexistence of identified microorganisms within the shared ecological niche of the gut, along with a similar response, whether involving an increase or decrease in relative abundance to environmental changes, especially the impact of running exercise. Consequently, we propose that a tendency for microbial taxa to positively correlate may indicate a cooperative, symbiotic relationship, while negative correlations may suggest an antagonistic relationship. Violin plots were generated using GraphPad Prism based on the data obtained from MicrobiomeAnalyst for each analysis. The network was constructed using iTOL (Letunic and Bork, 2021).

### 2.11 Taxon-function analysis prediction

We employed the web-based visualization application BURRITO (Browser Utility for Relating Microbiota Information on Taxonomy and function) (Mcnally et al., 2018) to predict the association between specific microbial taxa and functional activities in TRP metabolic pathways within the SED and RUN groups. The BURRITO framework is designed to uncover intricate relationships between microbial taxa and available attributes by integrating 16S RNA amplicon data or metagenomic data with KEGG functional annotations. Employing statistical modeling and machine learning techniques, BURRITO offers valuable insights into the taxonomic composition of microbial communities and their potential roles. Using BURRITO, we evaluated the proportion of the share for each taxa-based function’s total abundance attributed to TRP metabolism in each sample. For establishing taxonomy-function connections and estimating functional profiles, we utilized a taxonomic abundance table generated through QIITA as described above. Briefly, the 16S RNA amplicon data, underwent pre-processing, demultiplexing, and trimming to 250 bp. OTU picking employed a closed-reference approach, utilizing a default parameter set for taxonomy assignment, with a minimum similarity threshold set at 97%. We applied a custom KEGG BRITE database (Kanehisa et al., 2015) to establish the hierarchical relationship between functions associated with TRP metabolism, considering both taxonomic profiles and genomic content. The data, representing the total functional abundances of metabolic enzymes and taxon-function attributions for each sample, were downloaded from the BURRITO’s side-bar menu. Violin plots were constructed using GraphPad Software. To understand the dynamics and contributions of different taxonomic groups to specific functions, the changes in the total abundance of functions were assessed using the Mann–Whitney U test. Sankey network analysis visualized the average functional attributions for each taxon to the metabolic pathways associated with TRP metabolism. The Sankey diagram illustrates the average distribution of each function attributed to each taxon, offering an overview of the functional relationships within the microbial communities of the SED and RUN groups. The thickness of the arrows in the Sankey diagram represents the magnitude of taxon-specific attributions. Visualizations were generated using the Flourish tool Field (Seligman, 2011). Some figures incorporated icons created by BioRender.com and acknowledged in the figure legends.

## 3 Results and discussion

### 3.1 Changes in microbial diversity in the gut by running exercise

**Figure 1:**
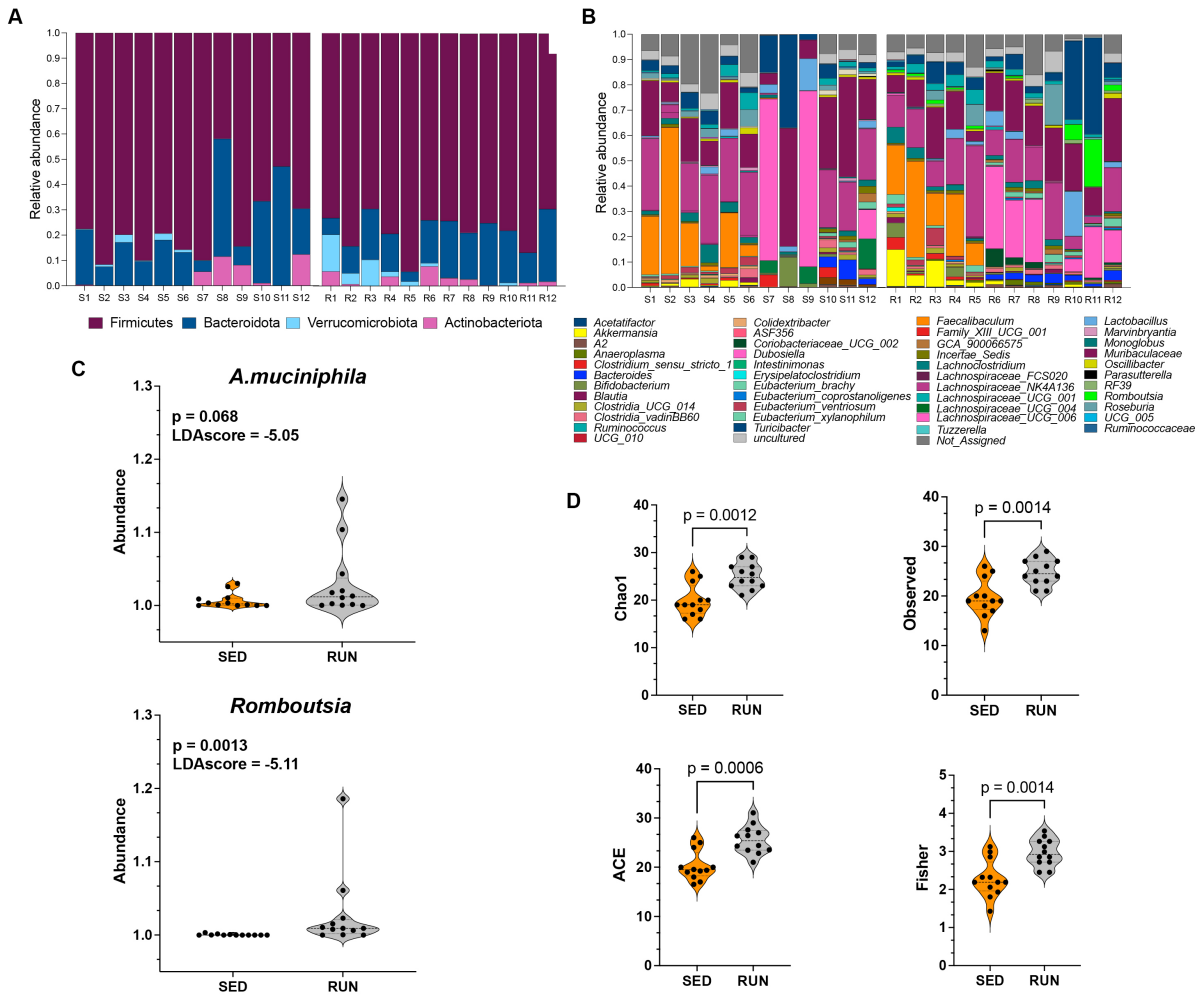
Taxonomic profiles and alpha diversity of bacterial communities in fecal samples from the SED and RUN groups. Panels **(A,B)** show the taxonomic profiles presented at the phylum level and genus level. Panel **(C)** shows linear discriminant analysis effect size (LEFsE) of bacterial communities from SED and RUN groups. The LEfSe box plots illustrate the log-transformed count level differences of *Romboutsia* and *A. muciniphila*, between the SED and RUN groups. Panel **(D)** depicts the alpha diversity indices, including Chao1, ACE, Observed, and Fisher Index, and the differences between groups were determined using the Mann–Whitney U test.

Data analyses were done using OTUs from the 24 fecal samples from SED (*n* = 12) and RUN (*n* = 12) groups. When examining the phylum levels, we found that engaging in running exercise led to higher levels of Firmicutes and decreased Bacteroidota (Figure 1A). Our data parallels other studies that have demonstrated an increase in the phylum Firmicutes and a decrease in Bacteroidetes by running exercise (Choi et al., 2013; Clarke et al., 2014; Kang et al., 2014; Yuan et al., 2022; Jin et al., 2023). The abundance of Actinobacteriota did not show changes between the groups (Figure 1A). Furthermore, we observed an elevated abundance of Verrucomicrobia in running mice, particularly *A. muciniphila* (Figures 1B,C), which aligns with findings from previous studies (Clarke et al., 2014; Jin et al., 2023), indicating that regular running exercise is associated with higher levels of *A. muciniphila*.

To further identify the features that played a pivotal role in distinguishing between the SED and RUN groups, we conducted LEfSe analysis. We found that *Romboutsia* (score of −5.11) and *A. muciniphila* (score of −5.05) were the most discriminative genera, as evidenced by exceptionally high LDA scores (Figure 1C). *A. muciniphila* is known to ameliorate host metabolic and intestinal homeostasis due to its components such as outer membrane proteins and extracellular vesicles. It can use mucin as its sole carbon, nitrogen, and energy source (Derrien et al., 2004). *A. muciniphila* produces acetate and propionate, which are short-chain fatty acids (SCFAs) linked to the regulation of body weight gain with anti-inflammatory and metabolic effects (Russell et al., 2013).

Alpha diversity analyses including richness (Observed, Chao 1), abundance-based coverage estimator (ACE, non-parametric), and Fisher diversity showed that samples from the RUN group had higher bacterial diversity (KW: Chao1, *p* = 0.002; Observed, *p* = 0.002; ACE, *p* = 0.001; Fisher, *p* = 0.002) compared to SED group (Figure 1D). Since the samples were obtained from the same environment, no notable differences were observed in the bacterial community structure, as confirmed by the beta diversity analysis (Permanova *p* = 0.5).

### 3.2 Running exercise modifies function-driven networks associated with TRP metabolism in the gut

**Figure 2:**
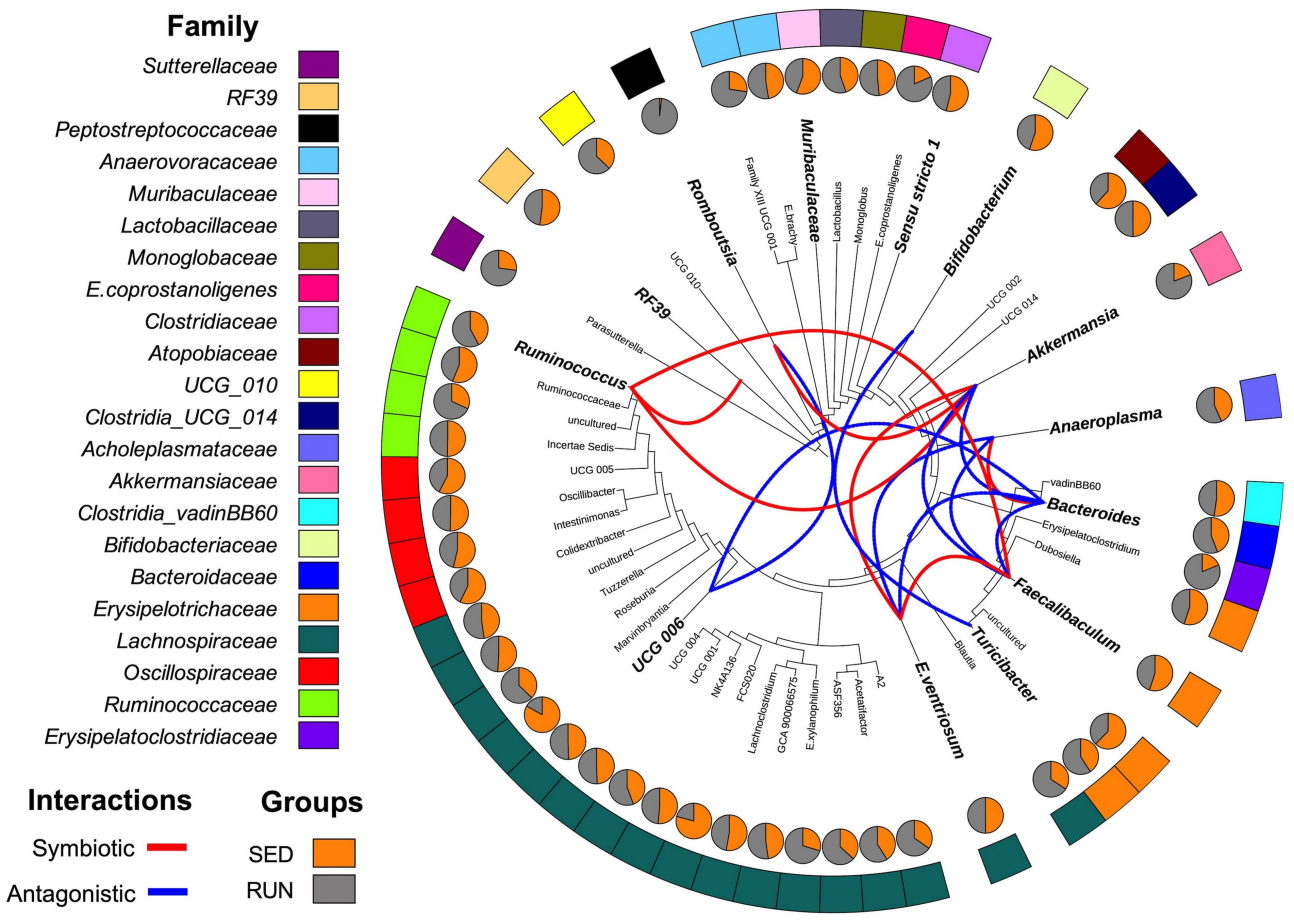
Diagrammatic phylogenetic tree illustrating the identified genera in fecal samples from the SED and RUN groups. The outer band represents the color-coded genera based on their respective families, and the inner pie charts display the merged relative abundances for each genus. The colored connections indicate the relationships between the genera as identified by the SparCC interaction network analysis. Red connections represent significant positive correlations (symbiotic interactions), while blue connections represent negative correlations (antagonistic interactions) at the genus level. Taxa highlighted in bold and displayed in larger font sizes indicate the taxa contributions to metabolic reactions as identified by BURRITO.

Through a BURRITO framework, we examined the potential correspondence between the identified changes in the bacterial interaction network and alterations in taxon-specific functions associated with TRP metabolism (Mcnally et al., 2018). It predicted that the functioning of various TRP enzymes associated with the kynurenine and indole pathways in the gut ecosystem of mice involves active contributions from several microbial taxa, including *Bacteroides, A. muciniphila, Bifidobacterium, Muribaculaceae, Turicibacter, Romboutsia, Ruminococcus, Anaeroplasma, RF39, UCG-006, C.sensu stricto 1, E.ventriosum*, and *Faecalibaculum*. To understand the interactions between these taxa in response to running exercise, we used a SparCC interaction network. We found changes in symbiotic (positive) and antagonistic (negative) relationships between microbial taxa in the gut ecosystem related to TRP metabolism. These relationships were represented by seven significant symbiotic correlations (indicated in red) and nine antagonistic correlations (indicated in blue) among the taxa at the genus level (Figure 2). *A. muciniphila* exhibited symbiotic relationships with *E. ventriosum*, *Ruminococcus*, and *Romboutsia*, while displaying antagonistic interactions with *Faecalibaculum* and *Bacteroides*. *Bacteroides*, in turn, developed a synergy with *Anaeroplasma* and antagonism with *E. ventriosum* and *Faecalibaculum*. *E. ventriosum* displayed a positive relationship with *Faecalibaculum*, which interacted positively with *Ruminococcus*. *Ruminococcus* positively interacted with *RF39*, while *UCG-006* interacted negatively with *Romboutsia* and *Bacteroides*. *Bifidobacterium* showed antagonism towards *Turicibacter*. Finally, *Muribaculaceae* and *C. sensu stricto 1* did not display any interactions in this network (Figure 2).

### 3.3 Running exercise induces changes in TRP degradation via the kynurenine pathway

We identified alterations in the network composition contributed to changes in the functional abundance of three key functions related to the utilization of TRP via the kynurenine pathway, including arylformamidase (kynB, K07130), catalase (CAT, K03781), and catalase-peroxidase (katG, K03782).

**Figure 3:**
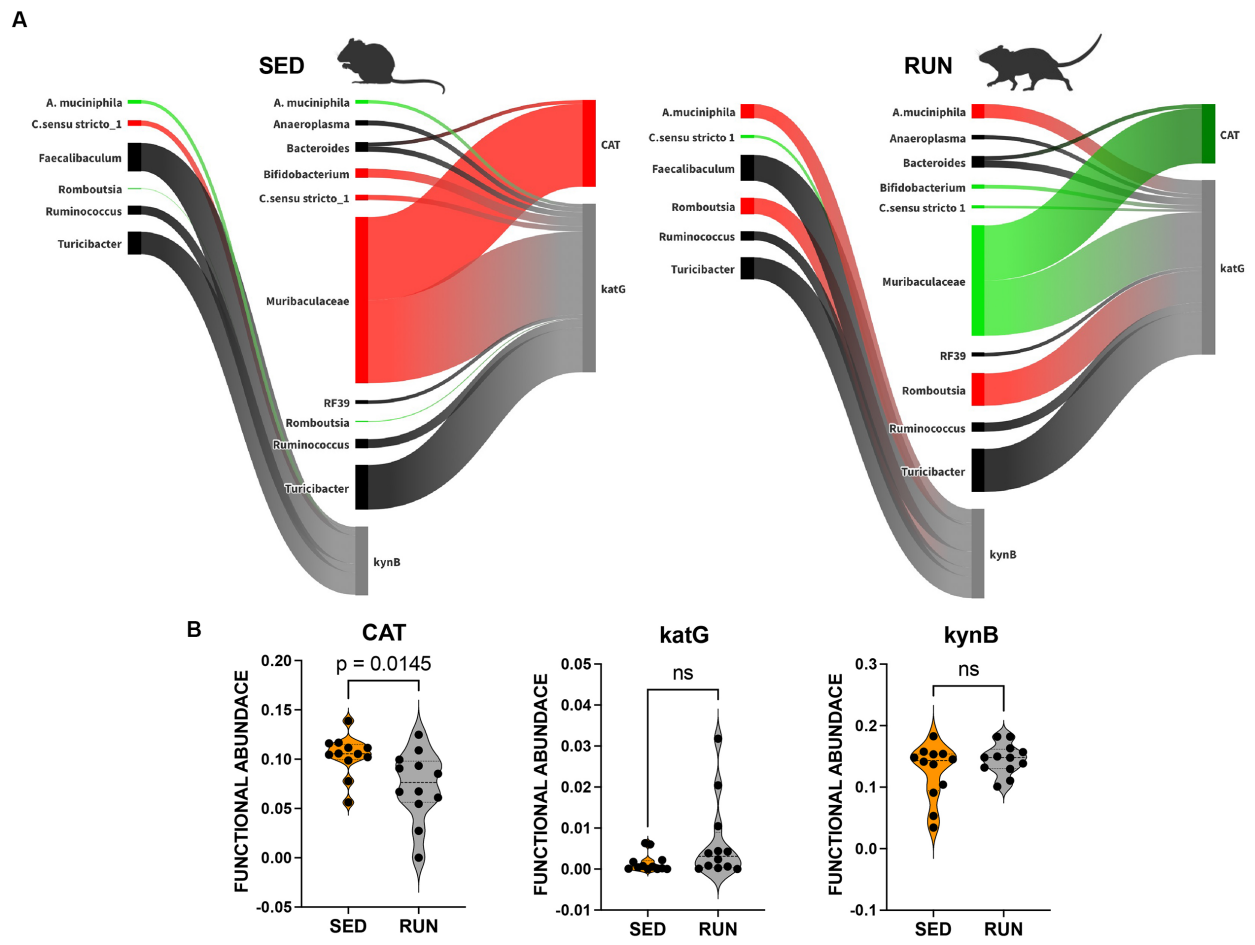
Sankey network illustrating the relationships between taxa and functions in the kynurenine pathway identified by BURRITO. Panel **(A)** illustrates the taxonomic and functional composition identified in the SED and RUN groups. The green color indicates a decrease in the magnitude of taxon-specific functional attributions, as depicted by the thickness of the arrows or functional abundance of the enzyme, while the red color indicates an increase. Black color – indicates no change. Panel **(B)** depicts violin plots displaying the differences between the SED and RUN groups in the functional abundance of identified enzymes involved in the kynurenine pathway. Statistical significance was determined using the Mann–Whitney U test. Sedentary and running mice icons were created with BioRender.com.

We found associations between *Turicibacter*, *C. sensu stricto 1*, *Romboutsia*, *Ruminococcus*, *Faecalibaculum*, and *A. muciniphila* with the functional abundance of kynB, an enzyme central to the synthesis of kynurenine and its derivatives within the kynurenine pathway (Figure 3A). Among these microbial community members, *A. muciniphila* and *Romboutsia*, which were found to have a symbiotic relationship (Figure 2), increased their contributions to the functional abundance of kynB, while *C. sensu stricto 1* decreased its contribution (Figure 3A). However, these changes did not significantly alter the functional abundance of kynB (Figure 3B). This observation suggests that the gut microbiota maintains homeostasis in response to perturbations induced by running exercise, ensuring the stability of the kynurenine pathway at a normal physiological level. We hypothesize that the shifts in the contributions of *A. muciniphila*, *Romboutsia*, and *C. sensu stricto 1* involve dynamic adjustments in the roles of other community members, collectively working to preserve the overall function.

**Figure 4:**
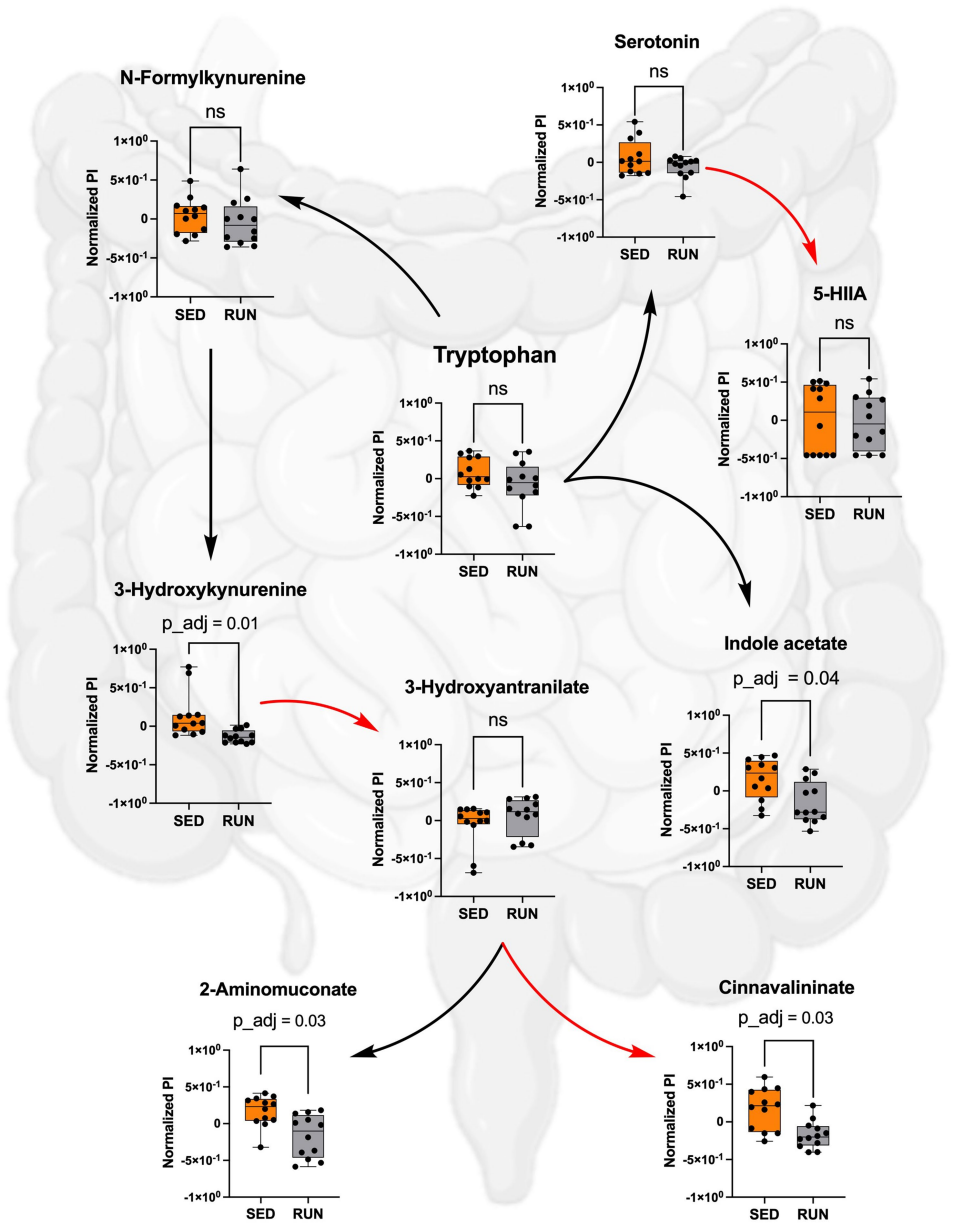
Metabolism of TRP in the SED and RUN groups through the kynurenine, indole, and serotonin pathways. The red arrows represent direct conversions between metabolites, while the black arrows indicate the presence of intermediates in the metabolite conversion. Additionally, the bar plots display the metabolic differences between the SED and RUN groups. Statistical significance was determined using the Mann–Whitney U test with FDR correction for multiple comparisons. The gut picture was created with BioRender.com.

Despite comparable TRP levels across groups, our analysis unveiled significant reductions in the downstream metabolites of the kynurenine pathway within the RUN group (Figure 4). Using the mummichog algorithm on the LC/MS data, we identified significant decreases in key empirical compounds that are TRP derivatives, including 3-hydroxykynurenine (*p*_adj = 0.01), cinnavalininate (*p*_adj = 0.03), and 2-aminomuconate (*p*_adj = 0.03). These predictions were validated using the Mann–Whitney U test, and the resulting *p* values were corrected for multiple comparisons using the FDR method (Figure 4).

We identified that both *Bacteroidetes* and *Muribaculaceae* contributed to the changes in functional abundance of CAT and katG, with *Muribaculaceae* being the most prominent contributor (Figure 3). Both enzymes catalyze the same reaction, the formation of cinnavalininate from 3-hydroxyantranilate (Figure 4). However, only CAT displayed a significant reduction in functional abundance (*p* = 0.0145) following running exercise, which correlated with the decrease in the share of *Muribaculaceae*.

In contrast, the decrease in shares of *Muribaculaceae* in the functional abundance of katG in the RUN group did not result in changes in its functional abundance. Moreover, we identified more contributors to the functional abundance of katG, including *Bifidobacterium*, *Bacteroides*, *Turicibacter*, *C. sensu stricto 1*, *Romboutsia*, *Ruminococcus*, *Anaeroplasma*, *RF39*, and *A. muciniphila* (Figure 3A). Although the bacterial interaction network revealed multiple symbiotic and antagonistic interactions among these taxa, including an increase in the shares of *Romboutsia* and *A. muciniphila* and a decrease in the contributions of *Bifidobacterium* and *C. sensu stricto 1* (Figure 2), no changes were observed in the functional abundance of katG in the RUN group (Figure 3B).

The effect of the decrease in shares of *Muribaculaceae*, *Bifidobacterium*, and *C. sensu stricto 1* on the functional abundance of CAT and katG following running exercise (Figure 3B) can be attributed to alterations in gut microbial diversity (Figures 1, 2), changes in bacterial metabolic preferences, and modifications in the gut environment. These modifications could include fluctuations in pH, oxygen levels, or nutrient availability (Mach and Fuster-Botella, 2017; Cella et al., 2021). These factors could have led to a diminished contribution from *Muribaculaceae*, *Bifidobacterium*, and *C. sensu stricto 1*, even if their relative abundance remained relatively stable.

### 3.4 Running exercise induces changes in TRP degradation via the indole pathway

**Figure 5:**
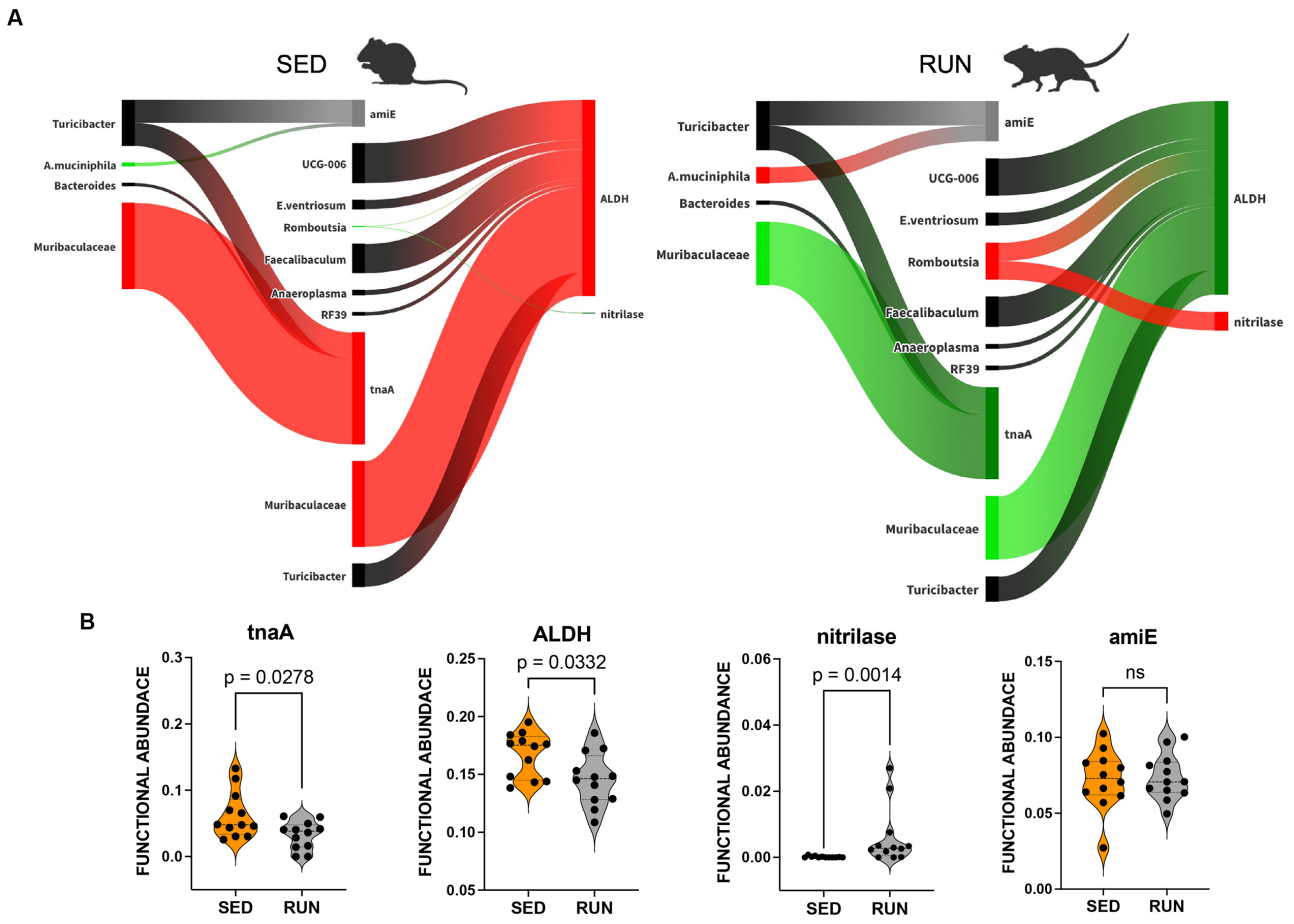
Sankey network illustrating the relationships between taxa and functions in the indole pathway identified by BURRITO. Panel **(A)** illustrates the taxonomic and functional composition identified in the SED and RUN groups. The green color indicates a decrease in the magnitude of taxon-specific functional attributions, as depicted by the thickness of the arrows or functional abundance of the enzyme, while the red color indicates an increase. Black color – indicates no change. Panel **(B)** depicts violin plots displaying the differences between the SED and RUN groups in the functional abundance of identified enzymes involved in the kynurenine pathway. Statistical significance was determined using the Mann–Whitney U test. Sedentary and running mice icons were generated via BioRender.com.

A modified bacterial interaction network, induced by running exercise, led to changes in the functional abundance of four key functions related to the utilization of TRP via indole pathway including tryptophanase (tnaA, K01667), aldehyde dehydrogenase (ALDH, K00128), nitrilase (K01501), and amidase (amiE, K01426). *Bacteroides*, *Muribaculaceae*, and *Turicibacter* were found to be associated with tnaA, a key enzyme involved in the conversion of TRP to indole (Figure 5A). A previous study has recognized *Bacteroides* as a contributor to the functional abundance of tnaA (Gao et al., 2020). However, our study identified the other bacterial species, including *Muribaculaceae* and *Turicibacter*, which demonstrated higher shares than *Bacteroides* for this metabolic reaction. Interestingly, the SparCC analysis revealed no specific relationships between these species (Figure 2). Moreover, engaging in running exercise reduced *Muribaculaceae’s* proportional contribution and significantly decreased tnaA functional abundance (*p* = 0.0278) despite the overall richness of *Muribaculaceae* remaining unchanged (Figure 5B). These findings suggest that *Muribaculaceae* may play a prominent role in contributing to the functionality of tnaA in the SED group. Conversely, *Bacteroides* and *Turicibacter* did not exhibit significant alterations in their shares to tnaA (Figure 5A). Running exercise may have triggered specific microbial interactions that led to a redistribution of functional activities of tnaA among the taxa-contributors. Thus, we speculate that the *Muribaculaceae* family underwent targeted metabolic adaptations in response to the running exercise, leading to decreased participation in this metabolic process and a consequent reduction in the functional abundance of tnaA.

Furthermore, we found that *Muribaculaceae*, *Turicibacter*, *UCG-006*, *E.ventriosum*, *Romboutsia*, *Faecalibaculum*, *Anaeroplasma*, and *RF39* were associated with the functional abundance of ALDH, an enzyme involved in various metabolic reactions, including TRP metabolism through the indole pathway (Figure 5A). ALDH is crucial in converting the TRP derivative indole acetaldehyde into indole acetate. Interestingly, we observed a significant reduction in the functional abundance of ALDH (*p* = 0.0332) in the RUN group (Figure 5B) correlated with the significant decrease in the content of indole acetate (*p*_adj = 0.04) in the fecal samples (Figure 4). Although the bacterial interaction network revealed multiple symbiotic and antagonistic interactions among *Roumboutsia, UCG-006*, *E. ventriosum*, *Faecalibaculum*, and *Anaeroplasma*, the functional abundance of ALDH was unaffected by the exercise regimen. At the same time, the decrease in *Muribaculaceae’s* share and the heightened contribution of *Romboutsia* to this metabolic reaction suggest that they may be particularly responsive to exercise-induced changes. Furthermore, *Romboutsia* was predicted as the sole taxon associated with an increase in the functional abundance of nitrilase, which is responsible for the conversion of 3-indoleacetonitrile to indole acetate (Figure 5A). Interestingly, a significant increase in *Romboutsia* richness in the RUN group correlated with a substantial rise in the functional abundance of nitrilase (*p* = 0.0014) (Figure 5B). This finding aligns with the previous study that demonstrated a significant positive correlation between *Romboutsia* and TRP in fecal samples obtained from physically active groups, as determined by Spearman’s correlation analysis (Tabone et al., 2021).

In addition, our analysis revealed that *Turicibacter* and *A. muciniphila* contributed to the functional abundance of amiE (Figure 5A), which is responsible for converting indole-3-acetamide to indole acetate. Prior studies have also highlighted the role of *A. muciniphila* in metabolizing TRP to indole acetate (Gao et al., 2018; Yin et al., 2021; Paeslack et al., 2022). Consistent with these findings, we observed an increase in the contribution of *A. muciniphila* to the functional abundance of amiE. However, the share of *Turicibacter* remained unchanged (Figure 5A). Notably, our analysis did not reveal any significant alterations in the functional abundance of amiE between the experimental groups (Figure 5B).

The observed increase in contributions of *Romboutsia’s* to the change in functional abundance of nitrilase and *A. muciniphila’s* contributions to amiE functional abundance (Figure 5A), hints at their potential function as a compensatory mechanism. This compensation may help counteract the reduction in indole acetate resulting from the decreased functionality of ALDH, ultimately maintaining indole acetate at a physiological level.

Taken together, these findings indicate that TRP degradation through the indole pathway in the RUN group underwent perturbations in the reactions responsible for producing indole (by tnaA) and indole acetate (by ALDH, nitrilase, and amiE). These changes were influenced mainly by the contributions of *Muribaculaceae*, *Romboutsia*, and *A. muciniphila*, which appeared sensitive to alterations in the community structure induced by exercise. This sensitivity led to specific metabolic adaptations within these taxa, contributing to the observed alterations in TRP degradation via the indole pathway. This adjustment could lead to an increased availability of tryptophan for transport to the brain, facilitating serotonin synthesis.

### 3.5 Running exercise promotes the transportation of TRP through the bloodstream, leading to elevated serotonin levels in the hippocampus and brainstem

It is recognized that engaging in running exercise can enhance blood flow throughout the body, potentially leading to an increased supply of TRP reaching various brain regions (Fernstrom and Fernstrom, 2006; Clark and Mach, 2016). Previous research has indicated that a sedentary and overindulgent lifestyle can impact the metabolic capacity of gut microbes, resulting in decreased circulating levels of TRP. This, in turn, leads to an increased metabolism of TRP in the gut, ultimately reducing the availability of TRP in the brain (O’mahony et al., 2015; Martin et al., 2018).

We further hypothesize that the observed reduction in the concentrations of identified TRP derivatives in the gut of the RUN group, potentially triggered by alterations in bacterial diversity and functional activity, could imply an elevated accessibility of TRP for transportation to the brain through the circulatory system. Once TRP enters the bloodstream, it must overcome the challenge of crossing the BBB to be taken up by the brain, which can contribute to serotonin synthesis (Höglund et al., 2019). As we mentioned earlier, sequestering TRP to produce serotonin, kynurenine, indole, and their derivatives in the gut could reduce TRP concentration in the blood, thereby limiting its availability to the brain. This reduction could constrain the availability of TRP to the brain, resulting in a potential decrease in serotonin production (Charnay and Léger, 2010; Gao et al., 2018; Waclawiková and El Aidy, 2018). It is crucial to note that the modulation of circulating TRP levels by the gut microbiota subsequently impacts serotonergic neurotransmission, which in turn influences the functioning of both the central and enteric nervous systems (O’mahony et al., 2015).

**Figure 6:**
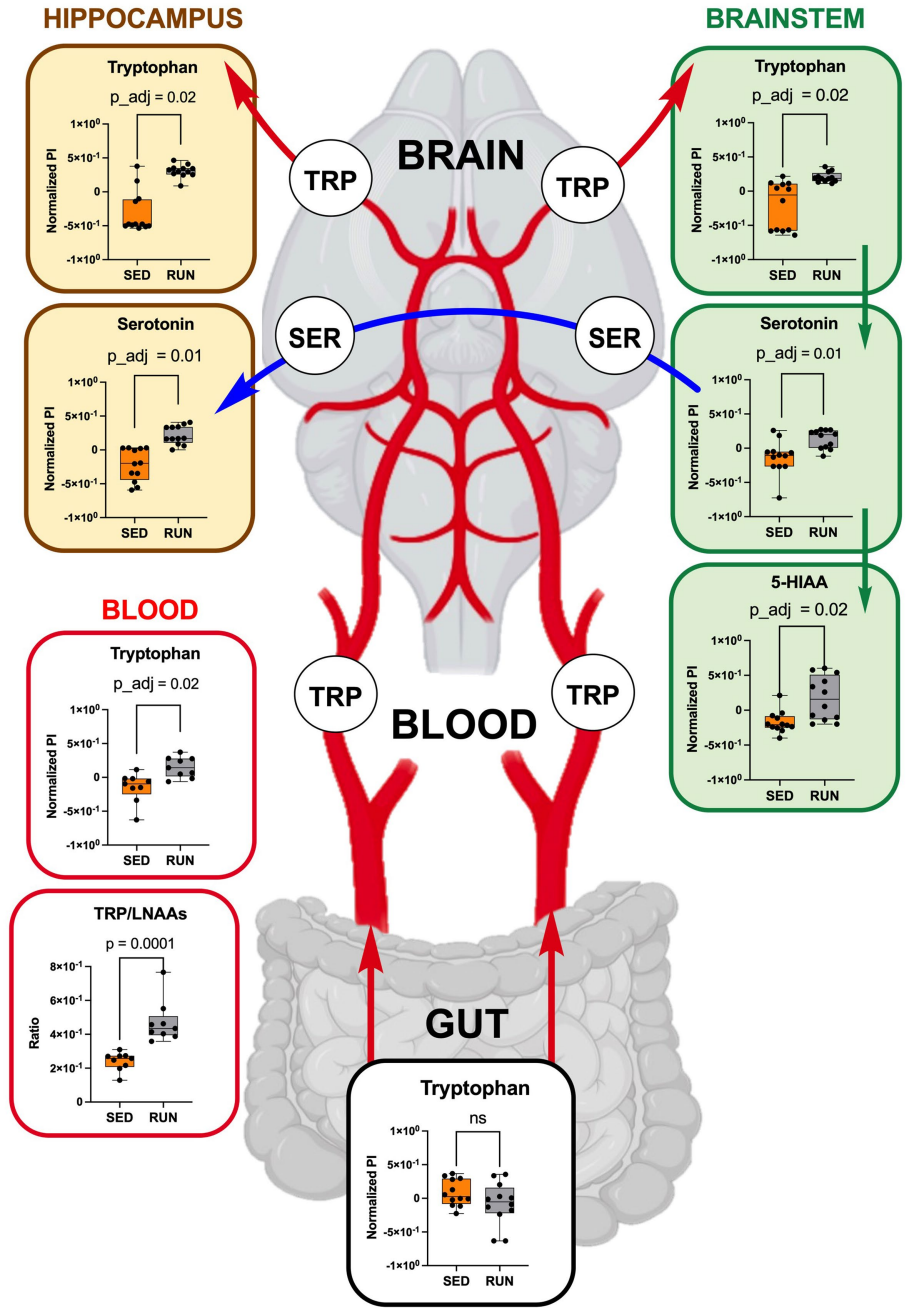
TRP metabolism along the gut-brain axis: focusing on TRP uptake from circulation (blood, each group *n* = 9) into the hippocampus and brainstem (each region and group, *n* = 12). Green arrows represent direct conversions between metabolites, red arrows indicate TRP transport from the gut via circulation to brain regions, and blue arrows represent the transport of serotonin through serotonergic fibers from the brainstem to the hippocampus. Bar plots illustrate metabolic differences between the SED and RUN groups. Statistical significance was determined using the Mann–Whitney U test with FDR correction for multiple comparisons. The TRP/LNAAs ratio was determined using the Mann–Whitney U test. The gut-blood-brain picture was created with BioRender.com.

To validate our hypothesis that running exercises facilitate TRP transport to the brain via circulation, we examined TRP levels in the blood, confirming higher TRP concentrations in blood samples from the RUN group compared to the SED group (*p*_adj = 0.02) (Figure 6). Subsequently, we calculated the TRP-to-large neutral amino acids (LNAA) ratio, encompassing isoleucine, valine, leucine, and phenylalanine, which serves as an indicator of the relative availability of TRP compared to other LNAAs in the bloodstream. The reason for calculating this ratio was to assess the potential impact of TRP availability on its uptake into the brain field (Leathwood, 1987). A high TRP/LNAA ratio indicates elevated TRP levels relative to other LNAA, potentially leading to increased TRP influx and enhanced serotonin synthesis in the brain. We observed a twofold elevation in the blood TRP/LNAA ratio (*p* = 0.0001), with the SED group ratio at 0.24 ± 0.02 and the RUN group ratio at 0.48 ± 0.04 (Figure 6). Therefore, our data supports the hypothesis that running exercise stimulates TRP transport to the brain through circulation.

Existing research highlights the positive impacts of exercise, particularly running, on the structure and function of the hippocampus, including the promotion of neurogenesis, leading to improved cognitive performance and mood (Chorna et al., 2013; Santos-Soto et al., 2013; Vivar and Van Praag, 2017; Rechavi et al., 2022). Importantly, discussions have emerged suggesting that serotonin, among other identified metabolites, might be a driving force behind hippocampal neurogenesis (Alenina and Klempin, 2015; Knobloch and Jessberger, 2017; Guo et al., 2023). Serotonin, synthesized by serotonergic neurons located in the brainstem’s raphe nuclei, is packaged into vesicles with the assistance of vesicular monoamine transporter 2 and then transported through serotonergic fibers to the hippocampus. In the hippocampus, it engages corresponding receptors and contributes to the regulation of neurogenesis, mood, cognition, and other functions (Anand and Dhikav, 2012; Alenina and Klempin, 2015; Dale et al., 2016; Livia Livint et al., 2023). Considering these insights, our study examined the TRP and serotonin levels in the hippocampus and brainstem of the SED and RUN groups.

Thus, by using GC/MS analysis, we found that running exercise led to a significant increase in TRP levels in the hippocampus (*p*_adj = 0.02) and brainstem (*p*_adj = 0.02) (Figure 6). Our results are consistent with previous research conducted in younger men, indicating that sustained exercise leads to notable enhancements in TRP availability in the brain in older men (Melancon et al., 2012). We next evaluated if the increase in TRP levels in the hippocampus and brainstem correlated with the increase in serotonin. The application of the mummichog algorithm to the LC/MS analysis, followed by the Mann–Whitney U test and FDR correction, identified a significant elevation in serotonin levels in both the hippocampus (*p*_adj = 0.01) and brainstem (*p*_adj = 0.01) of the RUN group (Figure 6). Thus, our findings are in line with other studies that have demonstrated an association between running exercise and elevated levels of serotonin in various brain regions, including the hippocampus and brainstem (Blomstrand et al., 1989; Chennaoui et al., 2000; Lin and Kuo, 2013; Klempin et al., 2018; Vecchio et al., 2018; Liu et al., 2019). For instance, a significant elevation in hippocampal serotonin levels was observed after 7 days of treadmill exercise (Chennaoui et al., 2000) and in the brainstem after 11 weeks of treadmill running 6 days a weekend (Blomstrand et al., 1989). Similarly, short-term voluntary running exercise for 6 days increased serotonin levels in the hippocampus (Klempin et al., 2018). Interestingly, a recent study demonstrated that oral administration of *A. muciniphila* had a biological effect on the induction of serotonin levels in the colon and hippocampus over a 5 weeks period (Yaghoubfar et al., 2020). However, the study’s authors have acknowledged that the reported effect is currently regarded as correlative, as no causative mechanisms have been identified. Next, the application of the mummichog algorithm to the LC/MS data revealed a significant increase in 5-HIAA levels (*p*_adj = 0.02) in the brainstem (Figure 6). This finding aligns with a previous study that reported elevated 5-HIAA levels in the brainstem following running exercise (Blomstrand et al., 1989). 5-HIAA serves as a crucial metabolite of serotonin, and changes in its ranks can provide valuable information about alterations in serotonin synthesis, metabolism, and neurotransmission, which are implicated in various physiological and pathological conditions. An increase in serotonin is typically accompanied by a corresponding rise in 5-HIAA levels, indicating enhanced serotonin synthesis and metabolism (Meeusen and De Meirleir, 1995). In line with this, our analysis suggests the absence of significant alterations in the levels of serotonin and its metabolite, 5-HIAA, in the gut (Figure 4), which indicates that the metabolism of TRP via the serotonin pathway in the gut occurs at normal physiological levels in both experimental groups.

### 3.6 The symbiotic interaction between *Romboutsia* and *A. muciniphila* triggered by running exercise is pivotal in regulating TRP metabolism along the microbiota-gut-brain axis

Importantly, taxon-function analysis revealed the prominent role of the taxon *Romboutsia* in modulating TRP metabolism along the microbiota-gut-brain axis. *Romboutsia* is a genus classified under the family *Peptostreptococcaceae*. Notably, sequences associated with the genus *Romboutsia* have predominantly been detected in samples derived from the intestines, with a primary focus on mammalian subjects (Gerritsen et al., 2019). As previously discussed, our LEfSe analyses identified *Romboutsia* as the most influential contributor to the differences in microbial composition between the SED and RUN groups. Notably, *Romboutsia* was found to be the sole contributor to the increased functional abundance of nitrilase (Figure 5). Based on these findings, we hypothesize that *Romboutsia* plays a pivotal role in modulating TRP metabolism, triggering a cascade of events that influence the overall microbial community and its functional activities. In this context, *Romboutsia* might indirectly impact the contributions of *Muribaculaceae* to the functional activities of ALDH and CAT, even without significant alterations in its taxonomic abundance. This influence can be attributed to various mechanisms, such as the microenvironment modulation or the disruption of ecological interactions within the microbial community.

Moreover, we observed a robust symbiotic relationship between *Romboutsia* and *A. muciniphila*, with both species showing increased contributions to the functional abundance of kynB and katG in the RUN group (Figure 3). Interestingly, despite this notable association, we did not observe any significant alterations in the functional abundance of these enzymes in our experiment. We further speculate that the strong symbiotic relationship between *Romboutsia* and *A. muciniphila* may play a crucial role in modulating the communication between the gut and the brain. This hypothesis is supported by previous studies that highlight the potential of *A. muciniphila* in modulating this communication and its beneficial effects in neurodegenerative diseases (Blacher et al., 2019; Ou et al., 2020; Cox et al., 2021; Yuan et al., 2022). Furthermore, there has been increasing recognition of the potential of *A. muciniphila*, a symbiotic bacterium residing in the intestinal mucosal layer, as a promising probiotic candidate. This recognition is due to its significant role in enhancing host metabolic functions and immune responses (Zhang et al., 2019).

We hypothesize that the combination of *Romboutsia* and its symbiotic partner *A. muciniphila*, both enriched in the gut through running exercise, have putative probiotic combinatorial effects. Their symbiotic interplay along the microbiota-gut-brain axis suggests a mutually beneficial relationship that influences TRP metabolism. However, due to limited available information, our understanding of the specific roles played by *Romboutsia* in their respective ecosystems still needs to be improved. Further investigation is needed to better understand the specific mechanisms involved in this symbiotic interplay.

## 4 Limitations of the study

While our study offers valuable insights into the connections among running exercise, gut microbiota, and TRP metabolism, it is imperative to acknowledge several limitations. The relatively small sample size of mice utilized in the study restricts the generalizability of the findings. Replicating the study with a larger sample size would enhance the robustness and reliability of the results. Additionally, employing an animal model, specifically mice, may introduce variations in gut microbiota composition and metabolism compared to humans. Moreover, the study focused on associations and correlations, and the observational nature of the study design precludes establishing causality. Beyond the mentioned limitations, it’s crucial to recognize constraints associated with the LC/MS method used for metabolite analysis in our study. Although LC/MS is potent for metabolite profiling, its limited structural information about detected metabolites can be addressed by incorporating LC/MS/MS analysis in future studies, providing more comprehensive insights into the changes in TRP metabolism induced by running exercise and the associated gut microbial alterations. Furthermore, we acknowledge that interpreting positive correlations as symbiotic and negative correlations as antagonistic is a hypothesis-generating step, providing a framework for further investigation. We recognize that additional experimental validation and functional analyses are necessary to confirm these relationships. Despite these limitations, our study contributes to the understanding of the intricate interplay between exercise, gut microbiota, and TRP metabolism, emphasizing the necessity for further research to address these limitations and expand knowledge in this field.

## 5 Conclusion

Our results substantiate the hypothesis that running exercise can effectively shape gut microbiota’s diversity and metabolic activity, inducing notable alterations in TRP metabolism along the microbiota-gut-brain axis. The identified symbiotic association between *Romboutsia* and *A. muciniphila* highlights the complex interactions between the gut microbiota and the brain, indicating their potential role in modifying the gut microenvironment and influencing TRP transport to the hippocampus and brainstem. To further explore the symbiotic association, we suggest employing a thorough microbiome profiling approach that integrates metagenomic and metatranscriptomic analyses. Additionally, incorporating a differential analysis to discern TRP metabolites originating from gut microbes and enterocytes would enhance our insights. Longitudinal studies and functional validation experiments, including behavioral assessments, are imperative for a comprehensive understanding of the intricate interplay between exercise, gut microbiota, and TRP metabolism along the microbiota-gut-brain axis. These findings could lay the foundation for advancing our understanding of the microbiota-gut-brain axis, providing guidance for future experiments and potential microbe-based strategies that contribute to developing diagnostic and therapeutic approaches for neurological diseases.

## Data availability statement

Microbiome and metadata are available in Qiita project number 15005, https://qiita.ucsd.edu/study/description/15005, and raw data is available at the European Bioinformatics Institute EBI accession: PRJEB66088; https://www.ebi.ac.uk/ena/browser/view/PRJEB66088. Metabolomics data are available at the NIH Common Fund’s National Metabolomics Data Repository (NMDR) website, the Metabolomics Workbench (Sud et al., 2016) https://www.metabolomicsworkbench.org. The Project ID: PR001823 (https://dx.doi.org/10.21228/M82Q61) includes two studies: ST002931 for GC/MS and ST002932 for LC/MS.

## Ethics statement

The animal study was approved by Institutional Animal Care and Use Committee of the Medical Sciences Campus at the University of Puerto Rico in compliance with the guidelines of the National Institutes of Health (NIH) for the care and use of laboratory animals. The study was conducted in accordance with the local legislation and institutional requirements. IACUC protocol number A660121.

## Author contributions

AV-M: Data curation, Formal analysis, Investigation, Writing – review & editing. NR-T: Investigation, Writing – review & editing. KA-R: Investigation, Writing – review & editing. FM-M: Investigation, Writing – review & editing. RA-M: Investigation, Writing – review & editing. GC-S: Investigation, Writing – review & editing. FG-V: Investigation, Conceptualization, Data curation, Funding acquisition, Methodology, Resources, Validation, Visualization, Writing – original draft, Writing – review & editing. NC: Conceptualization, Data curation, Funding acquisition, Investigation, Methodology, Resources, Validation, Visualization, Writing – original draft, Writing – review & editing, Formal analysis, Project administration, Supervision.
